# Factors associated with household food insecurity and depression in pregnant South African women from a low socio-economic setting: a cross-sectional study

**DOI:** 10.1007/s00127-018-1497-y

**Published:** 2018-02-14

**Authors:** Zulfa Abrahams, Crick Lund, Sally Field, Simone Honikman

**Affiliations:** 1 Department of Psychiatry and Mental Health, Perinatal Mental Health Project, Alan J Flisher Centre for Public Mental Health, University of Cape Town, Building B, 46 Sawkins Road, Rondebosch, Cape Town, 7700 South Africa; 2 Centre for Global Mental Health, Institute of Psychiatry, Psychology and Neuroscience, King’s College London, London, UK; 3 Department of Psychiatry and Mental Health, Alan J Flisher Centre for Public Mental Health, University of Cape Town, Cape Town, South Africa

**Keywords:** Food insecurity, Depression, Anxiety, Suicidality, Low-resource settings, Pregnant

## Abstract

**Purpose:**

Food insecurity has been linked with maternal depression in low-income settings. Few studies have looked at factors associated with both food insecurity and maternal depression as outcomes. This study aimed to assess factors associated with food insecurity and depression in a sample of pregnant South African women.

**Methods:**

We conducted a cross-sectional study at a Midwife Obstetric Unit in a low-income suburb in Cape Town. Pregnant women attending the clinic for their first antenatal visit were invited to participate. The shortened form of the US Household Food Security Survey Module was used to measure food insecurity. The Expanded Mini-International Neuropsychiatric Interview was used to diagnose depression, anxiety, alcohol and drug dependence, and assess for suicidal ideation and behaviour. Logistic regression modelling was conducted to explore factors associated with food insecurity and depression in separate models.

**Results:**

We found that 42% of households were food insecure and that 21% of participants were depressed (*N* = 376). The odds of being food insecure were increased in women with suicidal behaviour (OR = 5.34; 95% CI 1.26–22.57), with depression (4.27; 1.43–12.70) and in those with three or more children (3.79; 1.25–11.55). The odds of depression was greater in women who were food insecure (5.30; 1.63–17.30), substance dependent (15.83; 1.31–191.48) or diagnosed with an anxiety disorder (5.04; 1.71–14.82).

**Conclusions:**

Food insecurity and depression are strongly associated in pregnant women. The relationship between food insecurity and depression is complex and requires further investigation. Interventions that improve both food security and mental health during the perinatal period are likely to benefit the physical and mental well-being of mothers and children.

## Introduction

Despite the political and economic advances made in South Africa since achieving democracy in 1994, many South Africans continue to experience poverty and unemployment [1]. In a recent poverty report by Statistics South Africa [2], 30.4 million South Africans (55.5% of the population) were estimated to be living below the upper-bound poverty line, which is less than 992 South African Rands (ZAR992) or US$81.93 per person per month (pppm). South Africans living above the upper-bound poverty line are able to purchase both adequate levels of food and non-food items to ensure adequate health.

One of the key aspects of poverty is the inability to provide adequately for the households’ food needs. Food security is defined by the Food and Agriculture Organisation of the United States [3] as ‘a situation that exists when all people, at all times, have physical, social and economic access to sufficient, safe and nutritious food that meets their dietary needs and food preferences for an active and healthy life’. On the other hand, food insecurity exists when the availability of nutritionally adequate and safe foods, or the ability to acquire acceptable foods in an acceptable way, is limited or uncertain [4].

The food poverty line (FPL), is defined as the South African Rand value below which individuals are unable to purchase or consume enough food to supply them with the minimum per-capita-per-day energy requirements for adequate health [2], and is used to estimate the number of people living in extreme poverty. In South Africa, based on 2015 prices, the FPL was equivalent to R441 (US$36.42) pppm. In 2015, 25.2% of South Africans were living below the FPL, and were thus considered to be food insecure [2]. In addition, 41% of South African households are headed by women [1], giving women the decision making power, as well as the responsibility of being the breadwinner. During the perinatal period, women may be economically vulnerable, as their income-generating potential may have reduced, and their financial needs, such as having to eat well, attending regular clinic visits and providing childcare are likely to increase [5].

Research from low-income settings, including from within South Africa, shows that mental illness is associated with greater poverty, worse health, stressful interpersonal relationships and informal or no employment [6, 7]. During the perinatal period, the prevalence of depression is increased, particularly in low- and middle-income countries (LMIC) [8, 9].

Maternal depression is highly prevalent in low-income settings [8–11], and is associated with food insecurity [12–14]. Moreover, food insecurity is a strong predictor of maternal depression [15], while depression in mothers in low-income settings has been found to be a strong predictor of household food insecurity [16]. Patel [17] suggests that poverty and mental illness interact in a vicious cycle, whereby the risk of mental distress is increased in those living in poverty as a result of reduced social capital, social exclusion, violence and trauma, and poor access to health care. In turn, those with a mental illness are at an increased risk of poverty as a result of increased health expenses, lost employment, poor social support and stigma which surrounds mental illness [18]. While studies from LMICs such as Ethiopia [14, 19] and South Africa [15] have found that food insecurity is a strong predictor of maternal depression, studies showing that maternal depression predicts food insecurity were primarily in high-income countries such as Britain [16] and the USA [20]. Few studies [21] have looked at factors associated with both food insecurity and maternal depression as outcomes. Conducting these analyses in LMIC is important to understand more about the complex bidirectional relationship between food insecurity and depression. We hypothesised that, for pregnant women living in adverse settings, the poverty-mental illness cycle would be particularly prevalent. Therefore, this study aimed to use multivariate regression analysis to assess predictors of food insecurity and depression in a sample of pregnant women living in a low socio-economic setting in South Africa.

## Methods

### Participants

This cross-sectional study was conducted at the Hanover Park Midwife Obstetric Unit (MOU), in Cape Town, South Africa. Hanover Park is a low-income, residential suburb within the City of Cape Town, established in 1969 as part of the South African Apartheid governments Group Areas Act [22]. It has a population of approximately 45,000 people and experiences widespread gang activity and high rates of violent crimes. Coupled with this are numerous social problems such as school drop-outs, drug and alcohol abuse, prostitution, drug trafficking, and robberies. Education levels are low and unemployment is high. Only 21% of the residents have passed high school, while 36% are unemployed [23].

Every third woman, 18 years or older, attending the MOU for her first antenatal clinic visit, was invited to participate in the study between November 2011 and August 2012. Of the 559 eligible women who were invited to participate, 135 (24%) declined to participate, and 376 women were recruited. As the process was relatively time consuming, 48 women were not able to take the time to complete the questionnaires, due to work or childcare commitments.

### Testing procedures

An interviewer-administered socio-demographic questionnaire was used to collect data on participants’ age, obstetric information and feelings about pregnancy, level of education, relationship status, HIV status, socio-economic status, and prior experience of depression or anxiety.

Screening tools were selected based on either local validation data or their having been used in other resource constrained settings. The US Household Food Security Survey Module (HFSSM): 6-Item Short Form [4] was used to assess household food insecurity and hunger. The scale measures the frequency of running out of food, being unable to afford balanced meals, and skipping meals because of lack of food over the prior 6 months. Perceptions of social support from three possible sources (family, friends and a significant other) were measured using the Multidimensional Scale of Perceived Social Support (MSPSS) [24]. Threatening life experiences faced by women in the preceding 6 months were measured using the List of Threatening Experiences (LTE) [25]. The Revised Conflict Tactic Scales (CTS2) [26] was used to measure Intimate partner violence.

The Expanded Mini-International Neuropsychiatric Interview (MINI Plus) Version 5.0.0 [27, 28], was used to diagnose a major depressive episode (MDE) (Module A), generalised anxiety disorder (Module P), suicidality (Module C), alcohol dependence (Module K) and substance dependence (Module L), and has been validated in several countries, including South Africa [29–31]. The MINI was administered by an experienced, registered counsellor, who was supervised by a clinical psychologist. The MINI Plus is available in local South African languages—Afrikaans and isiXhosa [32]. All tools were administered in English, Afrikaans or isiXhosa, the languages spoken by the women attending the MOU.

Participants were provided with refreshments mid-way through the interview process. Participants were not provided with money for participation or transport. The testing procedures are explained in more detail published elsewhere [33].

### Ethical approval

Ethical approval for the study was obtained from the Human Research and Ethics Committee at the University of Cape Town (HREC REF: 131/2009). The Western Cape provincial Department of Health approved the use of the research site. All respondents who participated in the study provided written, informed consent after the procedure had been verbally explained to them. Consent forms were available in English, Afrikaans and isiXhosa. All those participants who were diagnosed with a mental disorder were offered on-site, free of charge counselling with a registered counsellor.

### Data analysis

Data analysis was carried out using STATA/SE statistical software package version 14.1 (StataCorp., College Station, TX, USA). Variables were described using frequency and percentages, and associations measured using Chi-square tests. An asset index was used to stratify households based on socio-economic status [34]. Asset indices have previously been used in studies in LMICs [35, 36]. To construct the asset index, information on ownership of electronic equipment (e.g., fridge or freezer, vacuum cleaner, television, microwave, washing machine, television), transport (owning a vehicle), sources of energy (electricity) and bank accounts (including credit card) were pooled together. Principal component analysis was used to stratify households into 4 quartiles representing least poor, poor, very poor and poorest.

The nine questions comprising Module C of the MINI Plus were used to develop three categories of suicidality based on experiences in the month prior to the interview. Suicidal ideation included questions on suicidal thoughts (questions c2–c4). Suicidal behaviour included questions on plans to commit suicide as well as suicide attempts (questions c5–c8). Suicidal ideation and behaviour referred to those who endorsed items pertaining to suicidal thoughts, and those who endorsed items pertaining to planning, preparing or attempting suicide (questions c2–c8).

The primary outcome variables in the regression analyses were; (1) food insecurity and (2) MDE. Currently there is no accepted measure or standardised way of measuring food insecurity in South Africa. The cut-points suggested in the user notes of the 6-item short form of the US Household Food Security Module [37] were used: a score of 0–1 indicates high or marginal food security; a score of 2–4 indicates low food security; a score of 5–6 indicates very low food security. We assigned food security status as follows: households with a score of 0–1 are referred to as food secure, while households with a score of 2–6 [derived by combining low food security (score of 2–4) and very low food security (score of 5–6)] are referred to as food insecure. MDE was diagnosed using Module A of the MINI Plus.

Model building techniques were used to develop multivariate logistic regression models for food insecurity and MDE separately. We controlled/adjusted for the following extraneous variables so as to exclude their effect on the outcome variable: participant income, household income, employment status, number of children, having an unplanned pregnancy, and feeling happy about the pregnancy. Univariate analysis was used to identify significant associations between food insecurity and a number of correlates. The following correlates, which were significantly associated with food insecurity, were then used to build the final multivariate model: participants’ income, employment status, household income, number of children, suicidal behaviour, MDE, anxiety disorder, history of mental illness, experience of a threatening life event in the past year, experiencing intimate partner violence, perceived social support, substance dependence and alcohol dependence.

In the second model, univariate analysis was used to identify significant associations between MDE and a number of correlates. The following correlates, which were significantly associated with MDE, were then used to build the final multivariate model: household income, number of children, unplanned pregnancy, feeling happy about pregnancy, substance dependence, anxiety disorder, food insecurity, experience of intimate partner violence, perceived social support, experience of a threatening life event in the past year, suicidal ideation and behaviour, history of mental illness, and alcohol dependence.

## Results

Participant characteristics are presented in Table 1. The study sample consisted of 376 pregnant women, of whom 158 (42%) were food insecure, 81 (21.4%) were diagnosed with MDE and 86 (22.8%) were diagnosed with anxiety disorder. Of those who were diagnosed with an MDE, 37% reported having a history of mental illness. Significantly more women who were food insecure had > 4 pregnancies (*p* = 0.005), > 3 children (*p* < 0.001), less education (*p* = 0.021), and less income (*p* = 0.049). More women who were food insecure had a history of mental illness (61.4 vs. 38.6%; *p* = 0.001), had experienced a threatening life event (58.2 vs. 41.8%; *p* < 0.001), and had been exposed to intimate partner violence (62.1 vs. 37.9%; *p* = 0.001), compared to those who were food secure.

**Table 1:** Demographic and social characteristics of participants by food security status

	Food secure	Food insecure	*p* value*
	*n* = 218 (58%)	*n* = 158 (42%)	
	*n* (%)	*n* (%)	
Age			
18–24 years	84 (57.5)	62 (42.5)	0.056
25–29 years	70 (61.4)	44 (38.6)	
30–35 years	45 (52.3)	41 (47.7)	
> 35 years	19 (63.3)	11 (36.7)	
Population group			
Black	72 (54.1)	61 (45.9)	0.164
Coloured	134 (59.8)	90 (40.2)	
White	5 (100)	0	
Indian	7 (50.0)	7 (50.0)	
Spoken language			
English	90 (64.8)	49 (35.2)	0.104
Afrikaans	63 (52.1)	58 (47.9)	
isiXhosa	65 (56.0)	51 (44.0)	
Gravidity			
1st Pregnancy	62 (64.6)	34 (35.4)	0.005
2–4 Pregnancies	144 (59.0)	100 (41.0)	
> 4 Pregnancies	12 (33.3)	24 (66.7)	
Parity			
No previous births	79 (64.7)	43 (35.3)	< 0.001
1 Previous birth	71 (55.5)	57 (44.5)	
2 Previous births	55 (66.3)	28 (33.7)	
> 3 Previous births	13 (30.2)	30 (69.8)	
Highest standard passed			
Grade 2–7	11 (52.4)	10 (47.6)	0.021
Grade 8–9	26 (42.6)	35 (57.4)	
Grade 10–12	181 (61.6)	113 (38.4)	
Employment status			
Employed	115 (68.5)	53 (31.5)	< 0.001
Unemployed	103 (49.5)	105 (50.5)	
Household income (monthly)			
R0–R500	3 (75.0)	1 (25.0)	0.049
R501–R1000	2 (33.3)	4 (6.7)	
R1001–R2000	16 (39.0)	25 (61.0)	
R2001–R5000	50 (54.4)	42 (45.6)	
> R5000	42 (67.7)	20 (32.3)	
Asset index			
Poorest	46 (48.9)	48 (51.1)	0.023
Very poor	49 (52.1)	45 (47.9)	
Poor	61 (63.5)	35 (36.5)	
Least poor	62 (68.1)	29 (31.9)	
Relationship type			
Married/stable partner	198 (58.6)	140 (41.4)	0.726
Casual/no partner	20 (55.6)	16 (44.4)	
Cohabiting with partner			
Yes	126 (60.3)	83 (39.7)	0.647
Sometimes	24 (54.5)	20 (45.5)	
No	54 (55.7)	43 (44.3)	
HIV status: positive	24 (58.5)	17 (41.5)	0.961
Planned pregnancy	88 (64.2)	49 (35.8)	0.064
History of mental illness	22 (38.6)	35 (61.4)	0.001
Experience of a threatening life event	61 (41.8)	85 (58.2)	< 0.001
Experience of intimate partner violence	22 (37.9)	36 (62.1)	0.001

*Chi-square test

Table 2 provides an overview of the six questions making up the HFSSM, and the number and proportion of households with positive answers to the questions. In 53% of households, the food that was bought did not last and there was not any money for more, while in almost half the homes (45%) household members couldn’t afford to eat balanced meals.

**Table 2:** Household food security questions

Questions	*n* (%)
Households where the food that was bought in the last 6 months sometimes/often did not last, and there was not any money to buy more	199 (52.9)
Households where, in the last 6 months, members often/sometimes could not afford to eat balanced meals	169 (45.1)
Households where, in the last 6 months, members ever had to cut down on the size of meals or skip meals because there was not enough money for food	76 (20.2)
Households where, in the last 6 months, members often cut down on the size of meals or skip meals because there was not enough money for food	58 (15.4)
Households where, in the last 6 months, members ever ate less than they felt they should because there was not enough money for food	77 (20.5)
Households where, in the last 6 months, members were ever hungry but did not eat because there was not enough money for food	46 (12.2)

Bivariate associations between food security status and common mental disorders and substance and alcohol dependence are shown in Table 3. Food insecurity was significantly associated with a diagnosis of MDE (*p* < 0.001), an anxiety disorder (*p* < 0.001), suicidal behaviour (*p* = 0.011), alcohol (*p* = 0.021) and substance dependence (*p* = 0.048).

**Table 3:** Bivariate associations between food security status and MINI-diagnosed disorders

	Food secure (*n* = 218)	Food insecure (*n* = 158)	*p* value*
	*n* (%)	*n* (%)	
Major depressive episode (MDE)	27 (33.3)	54 (66.7)	< 0.001
Any anxiety disorder	35 (40.7)	51 (59.3)	< 0.001
Suicidal ideation	28 (59.6)	19 (40.4)	0.813
Suicidal behaviour	8 (33.3)	16 (66.7)	0.011
Alcohol dependence	21 (42.9)	28 (57.1)	0.021
Substance dependence	5 (33.3)	10 (66.7)	0.048

*Chi-square test

The odds of food insecurity in pregnant women are shown in Table 4. In the full multivariate model, the odds of being food insecure was more than five times greater in women with suicidal behaviour [OR = 5.34 (1.26–22.5); *p* = 0.023], more than four times greater in women with a MDE [OR = 4.27 (1.43–12.70); *p* = 0.009], and almost four times greater in women with three or more children [OR = 3.79 (1.25–11.55); *p* = 0.019], when compared to women who are food secure. This model was able to correctly classify 74.2% of participants.

**Table 4:** Odds ratios for food insecurity using logistic regression

	Univariate		Multivariate	
	OR* (95% CI)	*p* value	OR* (95% CI)	*p* value
Participant income < R2000/month [ref: income > R2000/month]	1.96 (1.23–3.12)	0.004	1.48 (0.60–3.61)	0.394
Unemployment [ref: employed]	2.21 (1.44–3.40)	< 0.001	1.17 (0.51–2.69)	0.706
Household income < R2000/month [ref: income > R2000/month]	0.73 (0.53–0.99)	0.041	1.57 (0.67–3.66)	0.300
1 child [ref: no children]	1.16 (0.76–1.80)	0.479	1.43 (0.57–3.60)	0.444
2 children [ref: no children]	0.64 (0.38–1.06)	0.085	0.91 (0.34–2.43)	0.859
3 or more children [ref: no children]	3.70 (1.86–7.35)	< 0.001	3.79 (1.25–11.55)	0.019
Suicidal behaviour [ref: no suicidal behaviour]	2.96 (1.23–7.10)	0.015	5.34 (1.26–22.57)	0.023
Major depressive episode (MDE) [ref: no MDE episode]	3.67 (2.18–6.18)	< 0.001	4.27 (1.43–12.70)	0.009
Anxiety disorder [ref: no anxiety disorder]	2.49 (1.52–4.08)	< 0.001	1.13 (0.48–2.60)	0.779
History of mental illness [ref: no previous mental illness]	2.53 (1.42–4.52)	0.002	1.52 (0.59–3.91)	0.380
Threatening life event [ref: no threatening life events]	1.97 (1.30–3.01)	0.001	1.49 (0.75–2.97)	0.356
Intimate partner violence (IPV) [ref: no IPV]	2.63 (1.48–4.68)	0.001	1.26 (0.41–3.87)	0.685
Perceived social support [ref: minimal perceived social support]	0.97 (0.95–0.99)	0.002	0.98 (0.96–1.01)	0.277
Substance dependence [ref: no substance dependence]	3.40 (1.36–8.47)	0.009	1.57 (0.14–17.72)	0.716
Alcohol dependence [ref: not alcohol dependent]	2.02 (1.10–3.71)	0.023	1.36 (0.47–3.98)	0.571

*Odds ratio

The odds of having a MDE are shown in Table 5. In the full multivariate model, the odds of having a diagnosis of MDE was more than 15 times greater in women with substance dependence [OR = 15.83 (1.31-191.48); *p* = 0.030], five times greater in women with anxiety disorder [5.04 (1.71–14.82); *p* = 0.003], more than five times greater in women who were food insecure [5.30 (1.63–17.30); *p* = 0.006], and decreased with increasing perceived social support [0.95 (0.92–0.98); 0.003]. This model was able to correctly classify 88.7% of participants.

**Table 5:** Odds ratios for MDE using logistic regression

	Univariate		Multivariate	
	OR* (95% CI)	*p* value	OR* (95% CI)	*p* value
Household income < R2000/month [ref: income > R2000/month]	1.67 (0.76–3.64)	0.199	1.26 (0.42–3.71)	0.681
1 Child [ref: no children]	0.78 (0.46–1.33)	0.364	1.08 (0.22–5.39)	0.926
2 Children [ref: no children]	0.62 (0.32–1.19)	0.150	0.84 (0.15–4.73)	0.844
3 or more children [ref: no children]	2.86 (1.48–5.51)	0.002	1.75 (0.31–9.96)	0.529
Unplanned pregnancy [ref: planned pregnancy]	0.42 (0.23–0.74)	0.003	0.46 (0.14–1.53)	0.208
Happy about pregnancy [ref: unhappy about pregnancy]	1.91 (1.09–3.31)	0.023	0.99 (0.29–3.37)	0.988
Substance dependence [ref: no substance dependence]	4.52 (1.91–10.68)	0.001	15.83 (1.31–191.48)	0.030
Anxiety disorder [ref: no anxiety]	7.80 (4.51–13.51)	< 0.001	5.04 (1.71–14.82)	0.003
Food Insecurity [ref: food secure]	3.67 (2.18–6.18)	< 0.001	5.30 (1.63–17.30)	0.006
Intimate partner violence (IPV) [ref: no IPV]	2.94 (1.62–5.36)	< 0.001	2.76 (0.84–9.11)	0.096
Perceived social support [ref: poor social support]	0.95 (0.93–0.97)	< 0.001	0.95 (0.92–0.98)	0.003
Threatening life event [ref: no threatening life events]	4.79 (2.81–8.15)	< 0.001	1.62 (0.60–4.44)	0.343
Suicidal thoughts and behaviour (SIB) [ref: no SIB]	2.16 (1.21–3.85)	0.009	1.66 (0.55–5.03)	0.369
History of mental illness [ref: no previous mental illness]	5.84 (3.20–10.64)	< 0.001	1.96 (0.63–6.17)	0.247
Alcohol dependence [ref: not alcohol dependent]	1.97 (1.02–3.79)	0.043	0.61 (0.14–2.63)	0.505

*Odds ratio

## Discussion

In this cross-sectional study of pregnant South African women living in an urban, low socio-economic setting, we found that 42% of households were food insecure, and that 21% of participants were depressed. The bivariate analysis showed that food insecurity was significantly associated with all MINI-diagnosed disorders, except suicidal ideation. Using multiple logistic regression analysis, we found that the odds of being food insecure was increased in women with suicidal behaviour, with depression and in those with three or more children. In addition, we found that the odds of experiencing a MDE was greater in women who were food insecure, substance dependent or diagnosed with an anxiety disorder.

The 2016 Community Survey by Statistics South Africa [1] reported that 20% of South African households ran out of money to buy food in the past 12 months. Our study reported a much higher prevalence. In Hanover Park, 53% of pregnant women attending the MOU reported running out of food and not having any money to buy more. This high prevalence is not completely unexpected as Hanover Park is known for its high rates of poverty, unemployment and gangsterism [38], and food insecurity is a well-documented consequence of poverty and unemployment [39]. The relationship between poverty and food insecurity is complex and considered to operate in a vicious cycle. While poverty leads to hunger and lack of adequate nutrition; hunger and malnutrition decreases the ability to learn, work and adequately care for the family—all of which perpetuate poverty [40].

The association between poverty and common mental illnesses, as well as between food insecurity and common mental illnesses in LMIC are well-documented in systematic reviews [8, 41]. However, the size and direction of the association varies in different populations and by different measures of food insecurity and mental illness. In Equador, self-reported symptoms of depression in mothers increased the risk of the household being food insecure by almost three times [42], while the risk of food insecurity in Korean adults who reported feelings of sadness and despair were almost four times greater than in those who did not report feeling sadness and despair [43]. We found that the risk of being food insecure was 4.3 times greater in pregnant women who were diagnosed with depression, compared to those who were not depressed. This is much higher than the 8% increased risk of food insecurity that Pellowski et al. [44] reported, and the 5% increased risk that Dewing et al. [29] reported. While both studies took place in urban settings in South Africa, and used the EPDS to measure depression, Pellowski et al. used a sample of pregnant women with children, while Dewing et al. used a sample of postnatal women.

Our findings show that suicidal behaviour increases the odds of being food insecure by more than five times. While these results are similar to those of another South African study [29], our increased odds are far greater than the 12% increased risk previously reported. Furthermore, the relationship between food insecurity and suicidality appears to be bidirectional, with a study using a sample of non-pregnant adult women in India [45] and another using a sample of HIV positive adults in Uganda [46] showing that being food insecure increases the risk of suicidal thoughts and behaviour. Theories to explain the link include biological mechanisms whereby micronutrient deficiency and malnutrition have a negative impact on mental health, and psychological mechanisms whereby stress, extreme worry and anxiety cause maladaptive responses such as impaired thought processes, decision making and concentration, leading to suicidal ideation and behaviour [47]. As with other mental illnesses, suicidal behaviour may lead to increased health expenses, unemployment, social withdrawal and the inability to plan ahead, resulting in reduced ability to generate income or provide adequate amounts of food for the family [44].

Although many of the associations confirmed findings in the literature there was an exception. We found that having an anxiety disorder did not increase the odds of being food insecure. These results are unanticipated as food security is thought to cause uncertainty in the household, leading to feelings of stress that in turn lead to symptoms of anxiety and depression [48].

When examining the factors associated with depression, we found that the risk of depression increased fivefold with food insecurity or anxiety disorder. As we adjusted for household income (a proxy for poverty) in the multivariate regression model, we can conclude that the effect of food insecurity on MDE is independent of the effect of income. Similar findings have been reported before. Tsai et al. [15], using a longitudinal sample of pregnant South African women and adjusting for a number of extraneous variables including an asset wealth index which was used as a proxy for poverty, reported a dose–response relationship between food insecurity and greater depression symptom severity. However, in this large study, food insecurity was measured using a single-item question that referred to the number of days in the past week that the participant had gone hungry, whereas our study measured household food insecurity over the previous 6 months. Studies in urban [14] and rural [19] areas of Ethiopia reported that being pregnant and experiencing household food insecurity increased the risk of depression by more than four times.

Our findings suggest that the theory that common mental illnesses and poverty interact in a negative cycle [49, 50], can be extended to food insecurity and common mental illnesses. There are a number of potential explanations for the strong association observed between food insecurity and depression among the pregnant women in our study. Household food insecurity, especially in pregnant women may cause feelings of shame and desperation [48, 51], which result in decreased social engagement [52, 53], increased anxiety and distress, risk-taking behaviour, impulsivity, aggression [54], dysfunctional relationships, nutritional deficiencies, and symptoms of depression [54]. Conversely, mothers and prospective mothers who are depressed, may be at increased risk of remaining in poverty and being food insecure as a result of increased health expenditure, stigma [55], social withdrawal, lost work opportunities, risky behaviour, decreased earnings, and impaired ability to plan ahead [44, 56].

We found that the odds of MDE were 15 times greater in women who were substance dependent. This study confirms previous findings of a strong association between depression and substance dependence. In a systematic review by Lai et al. [57], all of the 18 studies used in the meta-analysis reported significant associations between drug abuse/dependence and depression. Similarly, Vythilingum et al. [58], reported a significant association between depression and substance use in a sample of pregnant South African women.

A strength of our study, in relation to previous work in this area, is the use of the MINI Plus assessment tool to diagnose depression, anxiety, alcohol and drug dependence and to assess for suicidal ideation and behaviour. In addition, the data collection was supervised by a clinical psychologist which strengthens the accuracy of the data. However, there are several limitations which need to be acknowledged. We do not have demographic information on 48 women who did not complete the questionnaires. We assessed food insecurity at a household level. As two-thirds of the participants had other children, intra-household food allocation would likely have favoured the children. It’s possible that the high levels of food insecurity reported by the mothers referred to their individual level of food insecurity, rather than accurately representing the household level. We used a shortened version of the HFSSM, which is only one of many tools used to measure food insecurity, making it difficult to compare our results to other studies. Furthermore, we used cut-points for the HFSSM developed in the US, which have not been validated in a South African population. The study design was cross-sectional, making it impossible to determine whether food insecurity preceded the mental illness, or the mental illness preceded food insecurity.

Further research is needed to improve our understanding of the relationships identified in this study and to determine the directions of causality. Intervention studies, using a randomised control study design, that combine poverty alleviation measures, such as food parcels or food vouchers with mental health interventions are needed. These studies would be most effective if they collect both qualitative and quantitative data.

Pregnancy is a particularly vulnerable time for women, as their income earning potential is decreased and their health and childcare needs are increased. Providing financial support to women during the perinatal period in the form of a pregnancy grant, as well as free and easily accessible mental health care is essential if we want to improve the health of mothers and their children.

## Conclusion

Our study provides important evidence of the strong associations between food insecurity, maternal depression and suicidality in low-income pregnant women in Cape Town. Longitudinal and qualitative data are required to assess potential causal relationships within these complex associations. Interventions aimed at alleviating food insecurity in pregnant women and new mothers are likely to benefit both their physical and mental well-being, thereby decreasing the growing burden of disease attributed to common mental illnesses.
